# Estimates of mortality attributable to influenza and RSV in the United States during 1997–2009 by influenza type or subtype, age, cause of death, and risk status

**DOI:** 10.1111/irv.12258

**Published:** 2014-06-27

**Authors:** Gonçalo Matias, Robert Taylor, François Haguinet, Cynthia Schuck-Paim, Roger Lustig, Vivek Shinde

**Affiliations:** a GlaxoSmithKline VaccinesWavre, Belgium; b Sage AnalyticaBethesda, MD, USA

**Keywords:** A/HINI, A/H3N2, influenza, mortality, respiratory broad, respiratory syncytial virus

## Abstract

**Background:**

Influenza and respiratory syncytial virus (RSV) cause substantial mortality from respiratory and other causes in the USA, especially among people aged 65 and older.

**Objectives:**

We estimated the influenza-attributable mortality and RSV-attributable mortality in the USA, stratified by age and risk status, using outcome definitions with different sensitivity and specificity.

**Methods:**

Influenza- and RSV-associated mortality was assessed from October 1997–March 2009 using multiple linear regression modeling on data obtained from designated government repositories.

**Results:**

The main outcomes and measures included mortality outcome definitions—pneumonia and influenza, respiratory broad, and cardiorespiratory disease. A seasonal average of 10 682 (2287–16 363), 19 100 (4862–29 245), and 28 169 (6797–42 316) deaths was attributed to influenza for pneumonia and influenza, respiratory broad, and cardiorespiratory outcome definitions, respectively. Corresponding values for RSV were 6211 (4584–8169), 11 300 (8546–14 244), and 17 199 (13 384–21 891), respectively. A/H3N2 accounted for seasonal average of 71% influenza-attributable deaths; influenza B accounted for most (51–95%) deaths during four seasons. Approximately 70% influenza-attributable deaths occurred in individuals ≥75 years, with increasing mortality for influenza A/H3N2 and B, but not A/H1N1. In children aged 0–4 years, an average of 97 deaths was attributed to influenza (A/H3N2 = 49, *B* = 33, A/H1N1 = 15) and 165 to respiratory broad outcome definition (RSV). Influenza-attributable mortality was 2·94-fold higher in high-risk individuals.

**Conclusions:**

Influenza-attributable mortality was highest in older and high-risk individuals and mortality in children was higher than reported in passive Centers for Disease Control and Prevention surveillance. Influenza B-attributable mortality was higher than A in four of 12 seasons. Our estimates represent an updated assessment of influenza-attributable mortality in the USA.

## Introduction

In the United States, annual wintertime influenza epidemics are associated with substantial numbers of medically attended outpatient visits, hospitalizations, and deaths.^1^–^3^ Estimates of influenza burden have been instrumental in increasing clinical awareness, informing vaccination policy, and guiding influenza prevention and control strategies. Influenza infections are often clinically under-recognized, not laboratory-confirmed when suspected, or difficult to confirm virologically because the virus is no longer detectable when patients present with complications of influenza (e.g., exacerbations of underlying cardiorespiratory disease). Consequently, conventional observational methods result in significant underestimates of influenza burden. Annual wintertime epidemics of respiratory syncytial virus (RSV) often coincide with influenza epidemics, and directly estimating the burden of RSV has presented the same challenges.

For decades, a variety of statistical multiple regression modeling techniques has been used by the United States (US) Centers for Disease Control and Prevention (CDC) and other groups to indirectly estimate the burden of influenza, including the burden attributable to secondary complications.^1^–^7^ The incidence of influenza-attributable disease outcomes has typically been estimated by statistically modeling the excess burden of respiratory, cardiorespiratory, or other clinical outcomes recorded during periods of influenza virus circulation over background rates of the same outcomes recorded outside these periods.^4^ More recently, modeling techniques that include pathogen-specific virological surveillance data [or International Classification of Disease (ICD)-coded outcomes data] as additional model variables have been used. These models allow for estimation of the burden of disease from multiple pathogens while simultaneously controlling for other potential disease drivers even during periods of influenza virus circulation.^2^,^5^,^6^

Detailed published estimates of influenza-attributable mortality by age, type, and subtype have not been updated by the CDC for seasons beyond the 2006–2007 influenza season.^3^ Because patterns of influenza virus circulation and the number of associated disease outcomes can change from decade to decade, updated burden estimates are needed to characterize changes in epidemiology. In addition, RSV is increasingly recognized as an important illness in elderly and high-risk adults, with a similar burden to non-pandemic influenza A.^8^ In designing the present study, we sought to generate updated and granular estimates of influenza- and RSV-attributable mortality in the USA according to six age strata, type, or subtype of influenza virus, and by high or low risk status. Moreover, because modeled excess mortality estimates may be attenuated for highly sensitive and less specific outcomes often used, such as cardiorespiratory mortality, we explored outcomes with a range of sensitivity and specificity. Those included a broader respiratory outcome definition (“respiratory broad”) which we hypothesized might provide a better trade-off between the under- or over-estimation of mortality associated with the classical pneumonia and influenza (P&I) or cardiorespiratory definitions, respectively.

## Methods

The objective of this retrospective database analysis was to estimate mortality attributable to influenza in the USA, stratified by age and risk status, for 12 influenza seasons (October 1, 1997 to March 31, 2009). Using multiple linear regression modeling, a proportion of wintertime mortality was attributed to circulating influenza viruses. The model utilized regional virology data to determine timing and level of circulating influenza virus types and subtypes. The analysis allowed assessment of the total influenza-attributable mortality in the USA and the relative contribution of various influenza types and subtypes.

### Virologic data

The weekly numbers of respiratory samples testing positive for influenza A and B types and subtypes in each of the four US census regions were obtained from the weekly influenza update, FluView, published by the CDC.^9^ These data are reported to the CDC via approximately 85 World Health Organization (WHO) collaborating laboratories and 60 National Respiratory and Enteric Virus Surveillance System (NREVSS) laboratories. Reports from both sources are combined, and the weekly total number of positive influenza tests, by virus type/subtype, and the percent of specimens testing positive for influenza are presented in FluView. For most of the study period, data were collected between week 40 of the first year of a season and up to and including week 20 of the second year. Weekly RSV surveillance data (counts of laboratory-confirmed RSVs regionally and nationally each week) were obtained from the NREVSS.^10^

### Mortality data

Mortality data were obtained from the US National Vital Statistics System (NVSS),^11^ and included demographic, geographic, and ICD-coded cause of death information. All records from subjects of any age were included. The period from April 2009 to September 2009 was excluded to avoid bias associated with the circulation of the 2009 A/H1N1 pandemic influenza virus strain. Records were excluded if they had data missing from the age, primary diagnosis, or month of death fields. Deaths were classified according to ICD codes; ICD9 codes were used before and during 2001 and ICD10 codes thereafter. ICD codes indicating both the underlying cause of death and any secondary diagnostic codes were captured. The 1999 transition from ICD-9 to ICD-10 was adjusted for by multiplying the monthly ICD-9 coded P&I rates by a constant defined by the summer 1999 rate divided by the summer 1998 rate;^12^ no adjustment was needed for the other two outcomes.

A variety of mortality outcome definitions were included, ranging from broad definitions to narrow ones. The broadest definitions were the most sensitive in terms of capturing influenza-attributable deaths, but lacked specificity. Narrower outcome definitions had a lower sensitivity and thus were unlikely to capture all influenza-attributable deaths, but were more specific to influenza, leading to a more conservative estimate of the influenza burden. Three mortality outcome definitions are reported here: (i) P&I; (ii) a novel, less specific outcome definition referred to as respiratory disease broadly defined, which included respiratory diseases, cough, breathing abnormalities, fever, and viral infections not otherwise specified (hereafter termed “respiratory broad”); and (iii) cardiorespiratory disease (Table S1).

### Statistical analysis

#### Preparation of time series data

Influenza and RSV virology time series were constructed by calculating, by type and subtype, the weekly percentages of positive tests out of the total number of influenza tests performed in each season. Mortality data were available only by month, while virology surveillance data were available by week. To deal with this, weekly values of mortality outcome counts were estimated by interpolation (SAS “Proc Expand” with spline). Each week's virus surveillance data were appended to the corresponding week's mortality outcomes. We investigated lagging the virologic data by 1, 2, and 3 weeks to mimic a delay in occurrence of death following influenza or RSV infection,^13^,^14^ but doing so did not improve model fit, as observed previously.^1^,^2^,^4^,^15^ Therefore, no lag was introduced.

#### Regression model

The program was run in SAS version 9.3. For each outcome definition, census region, age, and risk stratum, the same multiple linear regression model form was applied. There were six age groups: 0–4, 5–17, 18–49, 50–64, 65–74, and 75 + years. The risk status (high or low) of each subject was determined from co-morbidity diagnosis codes listed under cause of death. Co-morbidities considered included chronic obstructive pulmonary disease, cardiovascular disorders, kidney disorders, diabetes, immunosuppression, liver disorders, stroke, and central nervous system disorders; any mention of these disorders placed a person in the high-risk category; otherwise, they were considered low risk.

The model form used is given by the equation:


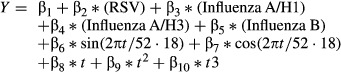


where *Y* is the incidence (rate) of outcome definition for each time period *t*, RSV and influenza are the proportion of laboratory isolates during *t*, sin and cos are harmonic functions of *t* (weeks), and the remaining terms track other types of secular trends in the data. The time period *t* used in the model is a running index of weeks starting October 1, 1997 and ending March 31, 2009. The RSV term controls for RSV epidemics.

The goodness of fit was assessed using adjusted *R*^2^; we found a substantial “lift” in the adjusted *R*^2^ value when introducing the virology terms into the base secular model. We did not adjust the model form (by including or excluding terms) individually for each outcome, age, and risk stratum, but applied the given form across all strata. Visual inspection showed that spikes during the winter-seasonal period were captured well by the model.

Incidence rates for each outcome definition per 100 000 population were calculated using census data; population denominators for the high- and low-risk groups were estimated using data from the National Health Information Survey. For each regional seasonal burden estimate and for each influenza type/subtype and for RSV, the 95% confidence interval (CI) was computed based on the standard error of the multiple regression model parameter for that pathogen. Specifically, each weekly point estimate was multiplied by the regression coefficient, and then, the weekly estimates were aggregated over the entire season. That procedure was repeated using the lower and upper 95% estimates for the regression parameter to obtain the seasonal upper and lower CIs for each regional estimate.

The national burden attributable to each pathogen term was determined by summing regional point estimates within each stratum. Because negative burden estimates are not biologically plausible, these estimates and their corresponding CIs were not included in the aggregations. A sensitivity analysis showed that marginally lower results were obtained when negative estimates were not discarded; for example, estimated seasonal influenza-attributed cardiorespiratory mortality decreased by 5% when negative estimates were included, and P&I mortality decreased by 9%.

National CIs (CI_national_) were obtained by pooling the variances of the regional estimates (σ^2^_region_) under the assumption of independence across regions, that is, σ_ij_ = 0 for regions i and j:


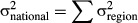


with all regional variances having the same denominator.


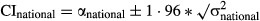


## Results

### Model fit

The model fit, as assessed by the adjusted *R*^2^, was generally good, especially for older age groups. For example, for the respiratory broad outcome, adjusted *R*^2^ ranged across regions from a high of 0·88 in the 75 + years age group to a low of 0·39 in the <2 years group. Moreover, in all cases, the addition of the virology terms to the model increased *R*^2^, usually by a substantial amount; again for the respiratory broad outcome, for example, adding the virology terms increased the adjusted *R*^2^ by 7–17%. Results were similar for the cardio-respiratory and P&I outcomes.

### Overall mortality

Using the P&I outcome definition, a seasonal average of 10 682 (standard deviation [SD]: 4476; min–max: 2287–16 363) deaths was attributed to influenza (Table1). For the respiratory broad and cardiorespiratory outcome definitions, the seasonal average of influenza-attributable deaths was 19 100 (SD: 7662; min–max: 4862–29 245) and 28 169 (SD: 11 120; min–max: 6797–42 316), respectively (Table1). The corresponding values for RSV-attributable deaths were 6211 (SD: 1116; min–max: 4584–8169), 11 300 (SD: 1639; min–max: 8546–14 244), and 17 199 (SD: 2631; min–max: 13 384–21 891) (Table1).

**Table 1:** Seasonal average mortality attributable to influenza and RSV (pneumonia and influenza, respiratory broad, and cardiorespiratory outcome definitions)

	Estimated number of deaths (influenza)				Estimated number of deaths (RSV)			
		
	Estimate	SD	Min–max	% of total	Estimate	SD	Min–max	% of total
P&I	10 682	4476	2287–16 363	5	6211	1116	4584–8169	3
Respiratory broad	19 100	7662	4862–29 245	3	11 300	1639	8546–14 244	2
Cardiorespiratory	28 169	11 120	6797–42 316	2	17 199	2631	13 384–21 891	1

P&I, Pneumonia and influenza; RSV, respiratory syncytial virus; SD, standard deviation.

The annual means of influenza-attributable mortality rates per 100 000 population were 3·70 (SD: 1·58; min–max: 0·75–5·58) for the P&I outcome definition, 6·61 (SD: 2·66; min–max: 1·59–9·98) for the respiratory broad outcome definition, and 9·76 (SD: 3·93; min–max: 2·23–14·45) for the cardiorespiratory outcome definition. Similarly, for RSV-attributable mortality, the annual mean rates per 100 000 population for the same outcome definitions were 2·15 (SD: 0·46; min–max: 1·55–2·90), 3·91 (SD: 0·65; min–max: 2·89–5·05), and 5·95 (SD: 1·1; min–max: 4·53–7·76).

The model attributed to influenza 5% of all P&I deaths, 3% of all respiratory broad deaths, and 2% of all cardiorespiratory deaths from 1997 through 2009 (Table1). Corresponding values for deaths attributed to RSV were 3%, 2%, and 1% (Table1).

### Mortality according to season and influenza type (respiratory broad outcome definition)

As expected, the number of deaths from influenza attributed by the model varied from season to season (Table2). The model estimated that the A/H3N2 virus accounted for the majority of influenza-attributable respiratory broad deaths, with a seasonal average of 13 545 deaths (71% of all such deaths; Table2); influenza B accounted for a seasonal average of 5478 deaths (29%), while the A/H1N1 subtype accounted for just 78 deaths (0·4%; Table2).

**Table 2:** Mortality attributable to influenza (respiratory broad outcome definition) by season, 1997–2009

	Estimated number of deaths (influenza)				% of total influenza		
			
	A/H1N1	A/H3N2	*B*	Total	Influenza A	Influenza B	Estimated number of deaths (RSV)
1997/1998	1	17 351	149	17 500	99	1	11 735
1998/1999	5	17 481	6673	24 159	72	28	12 006
1999/2000	11	21 599	182	21 792	99	1	14 244
2000/2001	206	341	11 303	11 850	5	95	12 469
2001/2002	10	22 256	4696	26 962	83	17	12 538
2002/2003	148	2495	9376	12 020	22	78	10 978
2003/2004	0	28 821	424	29 245	99	1	10 600
2004/2005	1	17 126	7032	24 159	71	29	8546
2005/2006	43	14 545	4869	19 458	75	25	8802
2006/2007	293	5145	5610	11 048	49	51	10 438
2007/2008	90	14 745	11 312	26 148	57	43	10 637
2008/2009*	125	632	4105	4862	16	84	12 606
Average season	78	13 545	5478	19 100	71	29	11 300

RSV, respiratory syncytial virus.

* Data included up to March 31, 2009.

During four of 12 influenza seasons in the study period, respiratory broad mortality attributable to influenza B was higher than that attributable to influenza A/H3N2. The model estimated that 95% of such deaths were attributable to influenza B in the 2000/2001 season, 78% in 2002/2003, 51% in 2006/2007, and 84% in 2008/2009 (Table2).

### Mortality according to age group, risk status, and influenza type

The distribution of influenza-attributable mortality across age groups was similar for the P&I, respiratory broad, and cardiorespiratory outcome definitions (Table3). Less than 1% of influenza-attributable deaths occurred in each of the 0–4 and 5–17 year age groups, with approximately 4%, 9%, and 14% of deaths in the 18–49, 50–64, and 65–74 year age groups for the cardiorespiratory outcome definition. The rates and numbers of death were highest in persons aged ≥ 75 years, accounting for 73% of all influenza-attributable deaths for the cardiorespiratory outcome definition. The risk of influenza-attributable death in the 75 + year age group relative to the 50–64 year age group was 27·8 for the P&I outcome definition, 22·7 for the respiratory broad outcome definition, and 22·2 for the cardiorespiratory outcome definition. The pattern of increasing mortality burden by age was consistent for influenza A/H3N2 and influenza B, but not for influenza A/H1N1 (Table3). Overall, there was a lower overall burden of H1N1 deaths in all age groups relative to deaths attributable to H3N2 and influenza B; however, deaths attributable to H1N1 were more common in children and younger adults relative to older adults (Table3).

**Table 3:** Seasonal average mortality attributable to influenza and RSV (pneumonia and influenza, respiratory broad, and cardiorespiratory outcome definitions) by age and influenza type and subtype

	Estimated number of deaths (influenza)					% of total influenza		Estimated number of deaths (RSV)	
			
	A/H1N1	A/H3N2	*B*	Total	Annual mean rate per 100 000	Influenza A	Influenza B	Total	Annual mean rate per 100 000
P&I									
0–4 years	10	29	17	56 (1%)	0·3	69	31	92 (1%)	0·5
5–17 years	12	23	19	54 (1%)	0·1	65	35	21 (0%)	0·0
18–49 years	43	288	146	476 (4%)	0·4	69	31	198 (3%)	0·2
50–64 years	0	578	226	804 (8%)	1·7	72	28	381 (6%)	0·8
65–74 years	0	873	241	1114 (10%)	5·9	78	22	805 (13%)	4·3
75 + years	0	6057	2121	8178 (77%)	47·5	74	26	4714 (76%)	27·4
All ages	65	7848	2769	10 682	3·7	74	26	6211	2·2
Respiratory broad									
0–4 years	15	49	33	97 (1%)	0·5	66	34	165 (1%)	0·8
5–17 years	19	30	34	84 (0%)	0·2	59	41	39 (0%)	0·1
18–49 years	31	433	279	743 (4%)	0·6	62	38	363 (3%)	0·3
50–64 years	13	1204	455	1672 (9%)	3·5	73	27	1060 (9%)	2·2
65–74 years	0	2003	660	2663 (14%)	14·1	75	25	1649 (15%)	8·7
75 + years	0	9826	4016	13 842 (72%)	80·1	71	29	8024 (71%)	46·4
All ages	78	13 545	5478	19 100	6·6	71	29	11 300	3·9
Cardiorespiratory									
0–4 years	22	52	30	105 (0%)	0·5	71	29	146 (1%)	0·7
5–17 years	21	39	41	101 (0%)	0·2	59	41	63 (0%)	0·1
18–49 years	99	669	319	1087 (4%)	0·8	71	29	867 (5%)	0·7
50–64 years	154	1847	509	2511 (9%)	5·4	80	20	1888 (11%)	4·0
65–74 years	4	3022	822	3848 (14%)	20·5	79	21	2483 (14%)	13·2
75 + years	191	14 765	5563	20 518 (73%)	119·0	73	27	11 753 (68%)	68·1
All ages	491	20 394	7285	28 169	9·8	74	26	17 199	6·0

P&I, pneumonia and influenza; RSV, respiratory syncytial virus.

In the 0–4 year age group, the risk of influenza-attributable death relative to RSV-attributable death was 0·6 for the P&I outcome definition, 0·6 for the respiratory broad outcome definition, and 0·7 for the cardiorespiratory outcome definition. Considering pediatric (<18 years) mortality overall in seasons where a comparable CDC estimate is available (i.e., 2004/05 through 2008/09), there was an annual average of 187 and 203 influenza-attributable deaths for the respiratory broad and cardiorespiratory outcome definitions, respectively (Table4).

**Table 4:** Seasonal average pediatric mortality attributable to influenza (respiratory broad and cardiorespiratory outcome definitions) and comparison with CDC estimates for seasons 2004–2005 through 2008–2009

Outcome definition	Estimated number (%) of deaths attributable to influenza A or B		

	Influenza A	Influenza B	Total
Respiratory broad (present study)	108 (58%)	79 (42%)	187
Cardiorespiratory (present study)	121 (60%)	82 (40%)	203
Laboratory-confirmed influenza in patients with febrile respiratory illness (notifiable) (CDC^14^)	54 (71%)	22 (29%)	76

CDC, centers for disease control.

As expected, influenza-attributable mortality was generally higher for high-risk versus low-risk individuals. In adults, the relative risk of death for high versus low risk status for the respiratory broad outcome definition ranged from 1·45 in individuals aged 18–49 years to 5·84 in those aged 65–74 years (Table5). Notably, the relative risk was higher in the 50–64 and 65–74 year age groups than in the 75 + year age group (Table5). In contrast, infants aged 0–4 years in the low risk group had a higher risk of influenza-attributable death versus those in the high-risk group, while children aged 5–17 years had an approximately equal risk regardless of risk status (Table5). A more marked difference between low-risk and high-risk groups for influenza A/H3N2 and B compared with A/H1N1 was observed (Table5).

**Table 5:** Seasonal average mortality attributable to influenza (respiratory broad outcome definition) by risk status and age

Age	Estimated number of deaths								
	
	A/H1N1		A/H3N2		*B*		Total		
				
	Low risk	High risk	Low risk	High risk	Low risk	High risk	Low risk	High risk	Ratio of high/low risk
0–4 years	11	5	36	12	25	11	72	28	0·38
5–17 years	12	8	19	11	9	25	40	44	1·09
18–49 years	15	19	169	265	119	154	303	439	1·45
50–64 years	0	12	243	967	107	326	351	1305	3·72
65–74 years	0	1	294	1709	91	541	385	2251	5·84
75 + years	0	0	2695	7166	990	2968	3685	10 134	2·75
All ages	38	45	3457	10 131	1342	4025	4837	14 201	2·94

## Discussion

This study has generated estimates of influenza-attributable mortality in the USA by influenza type and subtype and an estimate of RSV-attributable mortality. We estimated that between 1997 and 2009, an annual average of 19 100 deaths was attributable to influenza, and an annual average of 11 300 deaths was attributable to RSV, using the respiratory broad definition that included any mention of ICD9/ICD10 codes for any respiratory illness, cough, breathing abnormalities, fever, and other viral infections.

As expected, influenza-attributable mortality increased as outcome definitions became more sensitive: 10 682 deaths for the P&I outcome definition, 19 100 deaths for the respiratory broad outcome definition, and 28 169 deaths for the cardiorespiratory outcome definition. However, because influenza infections are likely to have a lower impact on mortality for more sensitive definitions, use of these outcome definitions can lead to mis-attribution of influenza-attributable deaths. By focusing on those deaths with any mention of respiratory illnesses (i.e., the respiratory broad definition), we sought to make a reasonable trade-off between sensitivity and specificity.

Our estimates are in broad agreement with CDC findings for the period 1976–2007, which estimated an annual average of 23 607 influenza-attributable deaths with underlying respiratory and circulatory causes and 6309 with underlying P&I causes.^3^ In contrast, our estimates were lower than earlier CDC estimates from 1990 to 1999 for influenza-attributable deaths with underlying respiratory and circulatory causes (36 155 deaths), but higher than CDC estimates for influenza-attributable deaths with underlying P&I causes (8097 deaths) during the same period.^2^ More recent estimates using a very different model approach to ours and that of the CDC attribute an average annual influenza-attributable death rate of 1·7 per 100 000 due to P&I causes between 1997 and 2007 in the USA,^16^ compared with our rate of 3·7 deaths per 100 000 for the P&I outcome definition between 1997 and 2009.

Modeled estimates of influenza-related mortality are the preferred source; however, estimates are tied to the modeling choices. Here, we used a modeling approach that included viral surveillance data representing the relative importance of each pathogen on a regional and weekly basis and, conservatively, a stationary trigonometric baseline to ensure that regular non-specific seasonal drivers of mortality (other pathogens) were considered. These choices are in line with those adopted in several previous studies^1^–^5^,^15^ and allow verification of the consistency of our estimates against previously published findings. However, the extent to which they are robust to model variations, such as the recent suggestion of different baseline functions (e.g., cubic spline) or other types of influenza incidence proxies, remains to be determined.^16^–^18^ Percent positive proxies were adopted in previous analyses^19^ but not all authors agree with this approach; it has been argued that regressing mortality linearly against the percent positive proxies may overestimate the contribution of influenza.^16^ Nonetheless, the observation that our estimates are largely consistent with epidemiological expectations regarding the attribution of burden to different age and risk strata, despite the use of the same model structure and virology time series in all cases, adds confidence to our results. It is worth noting that CDC estimates for influenza-attributable mortality for the same season have varied widely according to the study period, model used, resolution of outcomes, and inclusion or not of RSV virology.^2^,^3^,^7^ The most recent estimate from the CDC covering a common period with our study (1997–2007) reported an annual average of 32 805 influenza-attributable deaths with underlying respiratory and circulatory causes,^3^ while our study estimated an annual average of 21 861 influenza-attributable deaths using the cardiorespiratory outcome definition during the same period (data not shown). However, the CDC estimates did not account for the circulation of RSV.^3^

Centers for Disease Control estimates from 1990 to 1999 reported that mortality attributable to influenza B was higher than that attributable to influenza A/H3N2 only in one season (1990/1991).^2^ However, the present study, which covered the period 1997–2009, estimated that all-age mortality attributable to influenza B was higher than that of A/H3N2 in four seasons (2000/2001, 2002/2003, 2006/2007, and 2008/2009). The contrast between the studies is unsurprising, considering the lower circulation of influenza B in the 1990s compared with the first decade of the twenty-first century. In two seasons (2000/2001 and 2008/2009), the B Yamagata lineage strain circulated, while the B Victoria lineage strain, which appeared in the USA in 2001, circulated in the other two seasons. In these four seasons, between 51% (5610 deaths in 2006/2007) and 95% (11 303 in 2000/2001) of all influenza-attributable deaths were attributable to influenza B, reflecting the higher proportion of circulating influenza B in those seasons.

Since the unusually severe 2003/2004 Fujian A/H3N2 season, the US CDC has conducted national influenza mortality surveillance in children aged <18 years. During the five seasons from 2004/2005 to 2008/2009 for which the CDC surveillance and our study period overlapped, we attributed a seasonal average of 187 influenza deaths for the respiratory broad outcome definition and 203 deaths for the cardiorespiratory outcome definition in children aged <18 years. As expected, this was higher than the corresponding seasonal average of 76 laboratory-confirmed pediatric influenza deaths reported to the CDC surveillance system during this period,^20^ likely reflecting under-reporting associated with passive surveillance and its dependency on laboratory confirmation.

The mortality attributable to RSV was substantial in children aged <5 years, with approximately twice as many deaths attributed to RSV compared with influenza. This finding is in broad agreement with the 1990–1999 CDC estimate, which found that RSV-attributable P&I mortality was approximately 3·5-fold higher than influenza-attributable mortality in children aged <5 years.^2^ Our study also confirmed the recent recognition of the substantial mortality contribution of RSV in the elderly.^8^

We generated mortality estimates according to risk status, with high risk status defined as the presence of any underlying medical condition known to increase the risk of complications of influenza (e.g., chronic respiratory or cardiovascular diseases). In all but pediatric age groups, persons at high risk had higher influenza-attributable mortality rates than persons at low risk. Underlying health conditions have been previously shown to influence influenza-associated mortality.^18^ The greatest difference in mortality between high and low risk individuals was in the 65–74 year age group. There was less impact of risk status in adults aged 75 + years compared with those aged 50–74 years. A possible explanation is that frailty and susceptibility to influenza complications increase with age independently of underlying chronic conditions. The pattern in infants (0–4 years) was the opposite of that in adults, with most influenza-attributable mortality recorded in infants considered to be at low risk. This may be partially explained by the fact that infants have less time than adults to develop a high-risk chronic disease and have it recorded in their medical records.

The study had several limitations. First, mortality data were only available by month, and therefore, for seasons, when there was substantial overlap between the influenza and RSV epidemics, the model may not have ascertained the burden of each pathogen precisely. Second, although the relative distribution of influenza A, influenza B, and RSV is known to vary by age, all-age composite virologic data for influenza and RSV were used to guide the timing of the epidemic in the model. Finally, limitations inherent to indirect statistical approaches based on retrospective data must be considered. Although cyclic terms were included in the model to adjust for the putative influence of non-infectious factors and pathogens other than influenza and RSV on respiratory mortality, lack of specific information on the nature and patterns of other drivers of mortality adds uncertainty to the model estimates due to possible co-variability of these terms especially with the RSV term in the model. We should also note that, as in the analysis of any data series with temporal structure, *P*-values and confidence intervals are likely to be affected by autocorrelation in the residuals. Previous efforts have tried to circumvent this issue by including autoregressive terms as predictors in the model.^21^ However, this approach can blur the interpretation of the remaining regression coefficients, which then account only for the anomalies in the time series.^22^

These updated estimates of influenza and RSV mortality provide new insights into the changing burden of influenza and RSV during the last decade and critically inform public health policy decision-making on strategies to reduce this burden. These data also once again highlight the important burden of influenza in children, older adults, and at-risk groups and reinforce the rationale for public health measures to prevent and control influenza.

## Supporting Information

**Table S1.** Definition of mortality outcomes expressed in ICD9 and ICD10 codes.
