# Supplementary material for: Estimates of mortality attributable to influenza and RSV in the United States during 1997–2009 by influenza type or subtype, age, cause of death, and risk status
Source: Influenza Other Respir Viruses. 2014 Jun 27;8(5):507–15. doi: 10.1111/irv.12258 (PMC4181813; doi:10.1111/irv.12258)
Supplement: Supplementary file 1 — Table S1. Definition of mortality outcomes expressed in ICD9 and ICD10 codes. [file irv0008-0507-SD1.doc]

**Online-only supplement**

**Estimates of influenza-attributable mortality in the United States during 1997 – 2009 by type or subtype, age, and risk status**

Matias et al.

**Table S**1. Definition of mortality outcomes expressed in ICD9 and ICD10 codes

| **Outcome definition** | **ICD10 definition** | **ICD9 definition** |
| --- | --- | --- |
| Pneumonia and influenza | J09-18 | 480-488 |
| Respiratory broad | J00-99, B34, R05, R06, R50 | 460-519, 079, 786.0-786.4, 786.7-786.9 |
| Cardiorespiratory disease | I00-99, J00-99 | 390-519 |

ICD: International Classification of Diseases
